# Dextran Sulfate Sodium Salt-Induced Colitis Aggravates Gut Microbiota Dysbiosis and Liver Injury in Mice With Non-alcoholic Steatohepatitis

**DOI:** 10.3389/fmicb.2021.756299

**Published:** 2021-11-02

**Authors:** Bo Shen, Junjun Wang, Yuecheng Guo, Tianyi Gu, Zhenyang Shen, Cui Zhou, Binghang Li, Xianjun Xu, Fei Li, Qidi Zhang, Xiaobo Cai, Hui Dong, Lungen Lu

**Affiliations:** ^1^Department of Gastroenterology, Shanghai General Hospital, Shanghai Jiao Tong University School of Medicine, Shanghai, China; ^2^Shanghai Key Laboratory of Pancreatic Diseases, Shanghai General Hospital, Shanghai Jiao Tong University School of Medicine, Shanghai, China

**Keywords:** NASH, microbiota dysbiosis, colitis, liver fibrosis, inflammation

## Abstract

**Objective:** Inflammatory bowel disease (IBD) is characterized by gut microbiota dysbiosis, which is also frequently observed in patients with non-alcoholic fatty liver disease. Whether gut microbiota dysbiosis in IBD patients promotes the development of non-alcoholic steatohepatitis (NASH) remains unclear. We aimed to explore the role of gut microbiota dysbiosis in the development of NASH in mice with dextran sulfate sodium salt (DSS) induced colitis.

**Design:** Dextran sulfate sodium salt was used to induce colitis, and high fat (HF), in combination with a high-fructose diet, was used to induce NASH in C57BL/6J male mice. Mice were treated with (1%) DSS to induce colitis in cycles, and each cycle consisted of 7 days of DSS administration followed by a 10-day interval. The cycles were repeated throughout the experimental period of 19 weeks. Pathological alterations in colitis and NASH were validated by hematoxylin and eosin (H&E), oil red O, Sirius red staining, and immunofluorescence. Gut microbiota was examined by 16S rRNA sequencing, and gene expression profiles of hepatic non-parenchymal cells (NPCs) were detected by RNA sequencing.

**Results:** Dextran sulfate sodium salt administration enhanced the disruption of the gut–vascular barrier and aggravated hepatic inflammation and fibrosis in mice with NASH. DSS-induced colitis was accompanied by gut microbiota dysbiosis, characterized by alteration in the core microbiota composition. Compared with the HF group, the abundance of *p_Proteobacteria* and *g_Bacteroides* increased, while that of *f_S24-7* decreased in the DSS + HF mice. Specifically, gut microbiota dysbiosis was characterized by enrichment of lipopolysaccharide producing bacteria and decreased abundance of short-chain fatty acid-producing bacteria. Gene expression analysis of liver NPCs indicated that compared with the HF group, genes related to both inflammatory response and angiocrine signaling were altered in the DSS + HF group. The expression levels of inflammation-related and vascular development genes correlated significantly with the abundance of *p*_*Proteobacteria*, *g*_*Bacteroides*, or *f_S24-7* in the gut microbiota, implying that gut microbiota dysbiosis induced by DSS might aggravate hepatic inflammation and fibrosis by altering the gene expression in NPCs.

**Conclusion:** Dextran sulfate sodium salt-induced colitis may promote the progression of liver inflammation and fibrosis by inducing microbiota dysbiosis, which triggers an inflammatory response and disrupts angiocrine signaling in liver NPCs. The abundance of gut microbiota was associated with expression levels of inflammation-related genes in liver NPCs and may serve as a potential marker for the progression of NASH.

## Introduction

Non-alcoholic steatohepatitis (NASH) is an inflammatory form of non-alcoholic fatty liver disease (NAFLD). Progression of NASH results in fibrosis or even cirrhosis (Younossi et al., 2016), which has become the second most common cause of liver transplantation in the United States (Younossi et al., 2021). The prevalence of NAFLD in South Asia and Southeast Asia varies from 9 to 45% (Farrell et al., 2013). In China, it was estimated that there will be 32.61 million cases of NASH and 1.09 million cases of cirrhosis in 2030, imposing a significant public health burden (Estes et al., 2018). The pathogenesis of NASH can be explained by the “multiple hit hypothesis,” which considers risk factors, such as insulin resistance, hormones, nutritional factors, gut microbiota, and genetic factors (Buzzetti et al., 2016). Previous studies indicate that the gut microbiota plays an essential role in the development of NASH (Buzzetti et al., 2016; Leung et al., 2016; Lang and Schnabl, 2020). Inflammatory bowel disease (IBD), which has been correlated with the pathogenesis of NAFLD, is also characterized by intestinal bacterial overgrowth and imbalance (Cheng et al., 2018). Therefore, the relationship between NASH and IBD has received considerable attention.

Inflammatory bowel disease, comprising ulcerative colitis and Crohn’s disease, is often accompanied by NAFLD (Gisbert et al., 2007; Sartini et al., 2018). However, whether IBD can promote NASH development remains controversial. It has been reported that patients with IBD have a higher incidence of NAFLD (Magrì et al., 2019; Ritaccio et al., 2020). NAFLD accounts for up to 40.8% of the hepatic alterations in patients with IBD that do not have metabolic risk factors (Gisbert et al., 2007). Patients with severe IBD tend to show more severe liver steatosis compared with those with mild-to-moderate intestinal disease (Sartini et al., 2018). Intestinal inflammation induced by dextran sulfate sodium (DSS) is associated with hepatic inflammation and fibrogenesis in mice with NASH (Gäbele et al., 2011; Cheng et al., 2018). In contrast, IBD patients with NAFLD tend to have stable liver disease over 4–6 years, and the risk for progression of liver fibrosis is low, implying that IBD may not promote the progression of NAFLD (Ritaccio et al., 2020). Overall, the role of colitis-induced gut microbiota dysbiosis in NASH progression has not been thoroughly elucidated.

Studies have suggested that the gut microbiota is associated with NASH development (Aron-Wisnewsky et al., 2020). Microbe-derived metabolites such as short-chain fatty acids (SCFAs), which mainly include acetate, propionate, and butyrate may alleviate the progression of NASH (den Besten et al., 2015). Moreover, a previous study reported that SCFAs can reduce the hepatic aggregation of macrophages and proinflammatory responses (Deng et al., 2020). G-protein-coupled receptor 43 (GPR43) and GPR109A are the main receptors for SCFAs. GPR43 is mainly expressed in immune cells, such as neutrophils, eosinophils, dendritic cells, and monocytes, indicating its broad role in inflammatory and immune responses (Gasaly et al., 2021). GPR109A is also expressed by immune cells, such as neutrophils and macrophages, which play an important role in inflammatory responses (Li et al., 2018). Lipopolysaccharide (LPS), a component of the outer wall of Gram-negative bacteria, is known to promote NASH development by inducing macrophage activation and inflammation (Carpino et al., 2020). However, the relationship between LPS, SCFAs, or other microbial products and the expression of inflammation-related genes in the liver during NASH progression remains unclear.

In this study, we explored the association between DSS-induced gut microbiota dysbiosis and liver injury in mice with NASH. Mice maintained on a high-fat (HF) and -fructose diet, and DSS for 19 weeks were used to investigate the role of DSS-induced colitis in aggravating hepatic steatosis, inflammation, and fibrogenesis. Furthermore, 16S rRNA sequencing of the gut microbiota and RNA-seq of hepatic non-parenchymal cells (NPCs) was performed, and the potential impact of microbiota dysbiosis on gene expression of NPCs was explored. Collectively, our analysis may explain the mechanisms underlying aggravated liver injury in DSS-induced colitis mice.

## Materials and Methods

### Animal Models

Male C57BL/6J mice (Vital River Laboratory Animal, Beijing, China), at 9 weeks of age, were maintained under specific pathogen-free conditions at the Animal Resource Center of the Shanghai General Hospital. The protocols for *in vivo* experiments were approved by the Institutional Animal Care and Use Committee of the Shanghai General Hospital (No: 2019-A015-01).

### Experimental Design

A total of 27 C57BL/6J mice (male, 9 weeks old) were divided into three groups: (1) chow group (*n* = 9), which was administered with distilled water and standard chow; (2) HF group (*n* = 9), which was treated with a high-fructose diet [55% fructose (F0127, Sigma-Aldrich) + 45% sucrose (V900116, Sigma-Aldrich)] and 60% HF diet (D12492, Research Diets); and (3) DSS + HF group (*n* = 9), which was maintained on 1% DSS (MPbio.0216011080, MP Biomedicals), high-fructose diet (55% fructose + 45% sucrose), and 60% HF diet. DSS was administered in cycles, and each cycle consisted of 7 days of DSS administration followed by a 10-day interval with normal drinking water. The cycles were repeated throughout 19 weeks of experimentation.

The body weight of each mouse was monitored weekly throughout the period of experimentation. Stool, blood, liver, jejunum, ileum, and colon tissue samples were collected at week 19 and stored at −80°C until analysis.

### 16S rRNA Gene Analysis of Bacteria

A QIAamp PowerFecal DNA Kit (QIAGEN, Germany) was used to isolate DNA from stool samples. The V3–V4 hypervariable regions of the 16S rRNA gene were amplified using a two-step polymerase chain reaction (PCR) strategy, and sequencing was performed on an Illumina^®^ MiSeq^TM^ II platform at Shanghai South Gene Technology Company. Next-generation sequencing (NGS) reads for each sample were decoded using an in-house Java script, which was available upon request. Paired reads were assembled using Mothur, and sequences with ambiguous bases or length < 350 bp were removed for further analysis. Chimeric or contaminant sequences were also excluded, and the remaining high-quality sequences were grouped into operational taxonomic units (OTUs) with a threshold of 97% identity compared with the SILVA reference database. Taxonomy was assigned using the online software RDP classifier at the default parameter (80% threshold). Community richness, evenness, and alpha diversity analyses were analyzed using Mothur. The Bray–Curtis distance was used for performing the principal coordinate analysis (PCOA).

### Isolation of Non-parenchymal Cells From Mouse Liver

Non-parenchymal cells from mouse livers were isolated as per the instructions of the manufacturer provided with the Liver Dissociation Kit (Miltenyi Biotec, Germany). Hepatocytes were removed from the cell suspension by centrifugation at 50 × *g* for 2 min.

### RNA-Seq Analysis of Liver Non-parenchymal Cells

RNA was extracted from liver NPCs and sequenced by Sinotech Genomics (Shanghai, China). RNA integrity and purity were assessed using an Agilent Bioanalyzer 2100 (Agilent, United States) and quantified using a Qubit Fluorometer (Thermo Fisher, United States) and NanoDrop One (Thermo Fisher, United States). To be used for analysis, RNA samples had to meet the following quality criteria: RNA integrity number > 7.0, and 28S/18S ratio > 1.8. The following criteria were used to generate a heat map, which was used to analyze the count data: fold change > 2, *p*-value < 0.05. Read count data were imported into iDEP.93^1^ to analyze the differential expression of genes and pathways between the three groups (Ge et al., 2018). The R package in iDEP.93 was used for GO enrichment, DESeq, and generating Genewise clustering heatmaps.

### Real-Time Polymerase Chain Reaction Analyses of Inflammatory and Profibrogenic Factors Expressed in Liver Samples

Total RNA was extracted using the TRIzol Reagent (Takara Biotechnology, Dalian, China), and complementary DNA (cDNA) was synthesized using a PrimeScript RT reagent kit (Takara Biotechnology, Dalian, China). Real-time PCR was performed using Hieff quantitative polymerase chain reaction (qPCR) SYBR Green Master Mix (Yeasen, Shanghai, China) on a real-time PCR instrument (ViiA^TM^ Real-Time PCR Instruments, Thermo Fisher Scientific, United States). The results were analyzed using the 2^–ΔΔ*CT*^ method. All primers were synthesized by Xinbeibio (Shanghai, China). The primer sequences are as follows:

*Il1b:*
Forward primer: GAAATGCCACCTTTTGACAGTG
Reverse primer: TGGATGCTCTCATCAGGACAG
*Tnf*:
Forward primer: CCCTCACACTCAGATCATCTTCT
Reverse primer: GCTACGACGTGGGCTACAG
*Col1a1*:
Forward primer: TGAACGTGGTGTACAAGGTC
Reverse primer: CCATCTTTACCAGGAGAACCAT
*Acta2*, which encodes α-SMA
Forward primer: TGCTGGACTCTGGAGATGGTGTG
Reverse primer: CGGCAGTAGTCACGAAGGAATAGC
*Ffar2*, which encodes GPR43
Forward primer: CCACTGTATGGAGTGATCGCTG
Reverse primer: GGGTGAAGTTCTCGTAGCAGGT
*Hcar2*, which encodes GPR109a
Forward primer: CTGTTTCCACCTCAAGTCCTGG
Reverse primer: CATAGTTGTCCGTCAGGAACGG
*Tlr4*
Forward primer: AATGAGGACTGGGTGAGAAATGAG
Reverse primer: CTGTAGTGAAGGCAGAGGTGAAAG
*Il6*
Forward primer: TACCACTTCACAAGTCGGAGGC
Reverse primer: CTGCAAGTGCATCATCGTTGTTC
*Dll4*
Forward primer: GGGTCCAGTTATGCCTGCGAAT
Reverse primer: TTCGGCTTGGACCTCTGTTCAG
  *Pecam1*, which encodes CD31
Forward primer: CCAAAGCCAGTAGCATCATGGTC
Reverse primer: GGATGGTGAAGTTGGCTACAGG
*Tuba1a*, which encodes TUBULIN
Forward primer: GGCAGTGTTCGTAGACCTGGAA
Reverse primer: CTCCTTGCCAATGGTGTAGTGG

### Histological Analysis

The colon was cleaned with PBS and fixed for more than 24 h in 10% buffered formalin. After fixation, the samples were dehydrated and embedded in paraffin. Then, the jejunum, ileum, and colon were sectioned (5-μm thick). The samples were stained with hematoxylin and eosin (H&E) and examined by microscopy. The tissue cross-sections were graded from 0 to 3 for the level of inflammation, and the depth of inflammation was graded from 0 to 4.

Liver sections were processed routinely for H&E, oil red O, and Sirius red stainings to assess inflammation and steatosis. To detect extracellular matrix proteins, the slides were stained with 0.1% Sirius Red. Liver sections (10 fields per section) were graded by two blinded investigators (no foci = 0; <2 foci/field = 1; 2–4 foci/field = 2; >4 foci/field = 3) according to the method described by Kleiner et al. (Juluri et al., 2011). For liver steatosis and fibrosis, the area positive for the oil red O and Sirius red stainings was assessed.

### Immunofluorescent Staining of Formalin-Fixed Paraffin-Embedded Tissue

Formalin-fixed paraffin-embedded (FFPE) tissues were processed for staining with the following modifications of the method described previously (Gu et al., 2021). Following deparaffinization, antigen retrieval was performed in an antigen retrieval solution (Sangon, Shanghai, China) for 20 min at 125°C. Non-specific antibody binding was blocked with the immunostaining blocking buffer (Sangon, Shanghai, China), depending on the antibodies used. The primary antibodies were incubated overnight at 4°C. Sections were washed with PBS and Alexa Fluor-conjugated secondary antibodies (Invitrogen) for 1 h at 25°C. The nuclei were imaged using 4′,6-diamidin-2-fenilindolo (DAPI) counterstain (Yeason, Shanghai, China). The following primary antibodies specific to the following proteins were used: GFP (Abcam, Cambridge, MA, United States), α-SMA (Abcam), F4/80 (Abcam), CD31 (Abcam), PV-1 (Abcam), and ZO-1 (Abcam). Tissues were then incubated with the appropriate fluorophore-conjugated secondary antibodies (Cell Signaling Technology, CA, United States). Before imaging, nuclei were counterstained with DAPI. Confocal microscopy was performed using a Leica SP8 microscope with oil immersion objectives at ×40 magnification. The Fiji (ImageJ) software package was used for image analysis and fluorescence quantification.

### Statistical Analysis

Statistical analysis was performed using the IBM SPSS Statistics V19 software. Two-tailed Student’s *t-*test and Wilcoxon rank-sum test were performed as indicated. The Benjamini–Hochberg procedure (with BH-FDR correction) was applied in multiple testing for differentially expressed genes. The results were considered statistically significant at ^∗^*p* < 0.05; ^∗∗^*p* < 0.01.

## Results

### High-Fat Diet and High-Fructose Administration Induce Hepatocyte Steatosis

**FIGURE 1:**
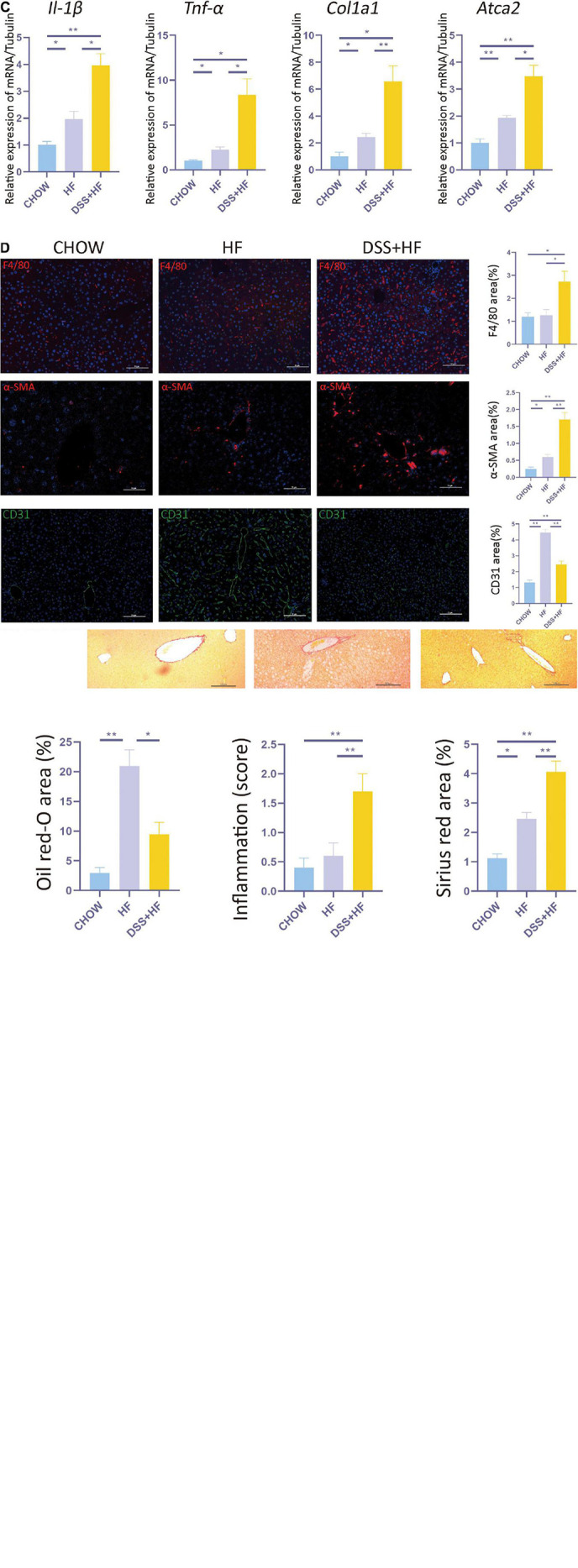
Dextran sulfate sodium salt (DSS) treatment aggravates hepatic inflammation and fibrosis in mice with non-alcoholic steatohepatitis (NASH). **(A)** Experimental model: After 1 week of acclimatization, 27 C57BL/6J mice (male, 9 weeks old) were divided into three groups: (1) chow group (*n* = 9), conventionally raised with distilled water and standard chow; (2) HF group (n = 9), raised with high-fructose diet and 60% high-fat diet; (3) DSS (+HF group (n = 9), raised with 1% DSS, high-fructose diet and 60% high-fat diet; DSS was applied in cycles and each cycle consisted of 7 days DSS administration followed by a 10-day inter-val with normal drinking water. The cycles were repeated throughout the experimental period of 18 weeks. **(B)** Represen-tative images for oil red, H&E and Sirius Red stained liver samples from chow, HF and DSS + HF group; The statistical analy-sis of the area of oil red, inflammation score and Sirius Red. The data are expressed as the mean ± SEM. *PP < 0.05; **PP <0.01. **(C)** *II-1*β, *Tnf*-α, *Col1a1*, and *ATCA2* expression at the mRNA level in liver samples from the above groups. **(D)** Representative immunofluorescence (IF) images of α-SMA, F4/80, and CD31 in liver samples from chow, HF and DSS + HF group; the statistical analysis of the staining area of α-SMA, F4/80, and CD31. The data are expressed as the mean ± SEM. *P < 0.05;**P < 0.01.

Three groups of mice, namely, chow, HF, and DSS + HF groups that were fed chow, HF diet, and high-fructose without or with DSS for 19 weeks, respectively, were included in this study (Figure 1A). The body weights of both the HF and DSS + HF groups were significantly higher than those of the chow group after weeks 12 and 13, respectively, while the DSS + HF group showed a significantly lower body weight than the HF group at week 12 (*p* < 0.01). HF diet led to an increase in body weight, but DSS administration prevented body weight gain in the DSS + HF group (*p* < 0.01; Supplementary Figure 1A). Oil red O staining indicated that the DSS + HF group exhibited more severe hepatocyte steatosis than the chow group (9.4 vs. 2.9%; *p* < 0.05), although it was less severe than that in the HF group (9.4 vs. 21.0%; *p* < 0.05; Figure 1B). Notably, the fat deposition pattern differed between the HF and DSS + HF groups, as shown by both, red oil O and H&E staining, with bullous lipidosis observed in the former and vesicular lipidosis in the latter group (Figure 1B).

### Dextran Sulfate Sodium Treatment Aggravates Hepatic Inflammation and Fibrosis

Compared with the chow and HF group, inflammatory cell infiltration in liver tissue increased significantly in the DSS + HF group (inflammatory score: DSS + HF 1.7 vs. chow 0.4; *p* < 0.01, DSS + HF 1.7 vs. HF 0.6; *p* < 0.01). In addition, we observed accumulation of necrotic tissue in the DSS + HF group, while a few scattered inflammatory cells were detected in the HF group (Figure 1B). Furthermore, expression levels of genes encoding proinflammatory cytokines IL-1β and TNF-α were significantly upregulated in the DSS + HF group compared with the chow or HF groups (Figure 1C; IL-1β: DSS + HF vs. chow; *p* < 0.01, DSS + HF vs. HF; *p* < 0.05) (TNF-α: DSS + HF vs. chow; *p* < 0.05, DSS + HF vs. HF; *p* < 0.05), which implied that hepatic inflammation was aggravated in mice with chronic colitis. F4/80 immunofluorescence staining was also performed to detect macrophages. Compared with the chow and HF groups, there was a significant increase in the F4/80-positive area in the DSS + HF group (DSS + HF 2.7% vs. chow 1.2%; *p* < 0.01, DSS + HF 2.7% vs. HF 1.3%; *p* < 0.05; Figure 1D). In addition, compared with the chow and HF groups, there was a significant decrease in the area positive for CD31 staining in the DSS + HF group (DSS + HF 2.6% vs. chow 1.3%; *p* < 0.01, DSS + HF 2.6% vs. HF 4.7%; *p* < 0.01; Figure 1D).

In addition to hepatic inflammation, the degree of liver fibrosis was evaluated using Sirius red staining. Compared with the chow and HF groups, a significant increase in the severity of liver fibrosis was observed in the DSS + HF group (DSS + HF 4.1% vs. chow 1.1%; *p* < 0.01, DSS + HF 4.1% vs. HF 2.6%; *p* < 0.05; Figure 1B), which was consistent with the degree of hepatic inflammation. The degree of liver fibrosis was significantly higher in the HF group compared with the chow group (HF 2.5% vs. chow 1.3%; *p* < 0.05). We next used the Metavir scoring system to assess the degree of fibrosis. In the chow group, almost no fibrosis was detected, which was therefore scored as F0. However, in the HF group, the fibrosis was accompanied with the expansion of most portal zones and was scored as F1. In the DSS + HF group, presence of fibrosis, expansion of most portal zones, and occasional bridging led to the score of F2 (Figure 1B). Furthermore, we detected expression of profibrogenic genes, namely, α-SMA and *Col1a1*. The results showed that α-SMA and *Col1a1* mRNA levels were significantly increased in the DSS + HF group (α-SMA: DSS + HF vs. chow; *p* < 0.01, DSS + HF vs. HF; *p* < 0.05) (*Col1a1*: DSS + HF vs. chow; *p* < 0.05, DSS + HF vs. HF; *p* < 0.01; Figure 1C). The expression of α-SMA protein was detected using immunofluorescence. There was little fluorescence detected in the chow group. In contrast, samples from mice on the HF diet showed moderate α-SMA-positive staining, which was enhanced by DSS treatment (DSS + HF vs. chow; *p* < 0.01, DSS + HF vs. HF; *p* < 0.01; Figure 1D).

### Dextran Sulfate Sodium Administration Enhances Intestinal Inflammation and Promotes Disruption of the Gut–Vascular and Intestinal Barrier in Mice With Non-alcoholic Steatohepatitis

**FIGURE 2:**
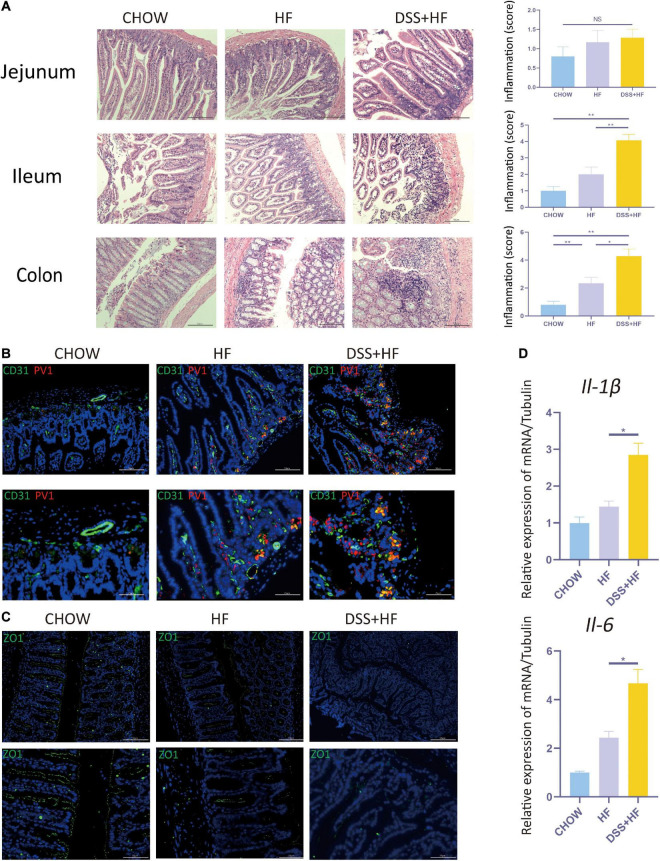
Dextran sulfate sodium salt administration enhances intestinal inflammation and the disruption of gut-vascular barrier and intestinal barrier in mice with NASH. **(A)** Representative images of jejunum, ileum, and colon in chow, HF, and DSS + HF group; The statistical analysis of inflammation score. The data are expressed as the mean ± SEM. NS, not statistically significant; *P < 0.05; **P < 0.01. **(B)** Representative IF images indicating co-localization of PVl (red) with CD31 (green) in colon from chow, HF, and DSS + HF group. **(C)** Representative IF images of Z0-1 (green). **(D)** *II-1*β and *II-6* expression at the mRNA level in colon from the above groups. *P < 0.05.

The DSS + HF group exhibited more severe epithelial damage and higher levels of leukocyte infiltration in the ileum and colon, but not in the jejunum. In the ileum, the inflammation score of the DSS + HF group was significantly higher than that of the HF and chow groups (DSS + HF vs. chow; *p* < 0.01, DSS + HF vs. HF; *p* < 0.01). Additionally, the inflammation score of the DSS + HF group was significantly higher than that of the HF and chow groups (DSS + HF vs. chow; *p* < 0.01, DSS + HF vs. HF; *p* < 0.05; Figure 2A). Next, compared with the HF group, IL-6 and IL-1β mRNA levels were increased in the colon of mice in the DSS + HF group (IL-6: DSS + HF vs. HF; *p* < 0.05) (IL-1β: DSS + HF vs. HF; *p* < 0.05; Figure 2D).

Very few co-localization of CD31 and PV1 was detectable in the chow group. However, in the HF group, there was a small degree of co-localization between CD31 and PV1, with the co-localization area being mainly located near the basement membrane of the colon. Compared with the HF and chow groups, the degree of CD31 and PV1 colocalization was high in the DSS + HF group (Figure 2B). These results suggest an increase in the vascular mucosal permeability in the HF group. Moreover, this increase in vascular mucosal permeability was more severe in the DSS + HF group.

ZO-1 immunofluorescence was analyzed to detect the permeability of the intestinal mucosa. Almost no staining was detected in the DSS + HF group. In contrast, increased ZO-1 staining was observed in the chow and HF groups. These results suggest that the permeability of the intestinal mucosa was severely damaged in the DSS + HF group (Figure 2C).

### Dextran Sulfate Sodium Treatment Alters the Gut Microbiota Composition

**FIGURE 3:**
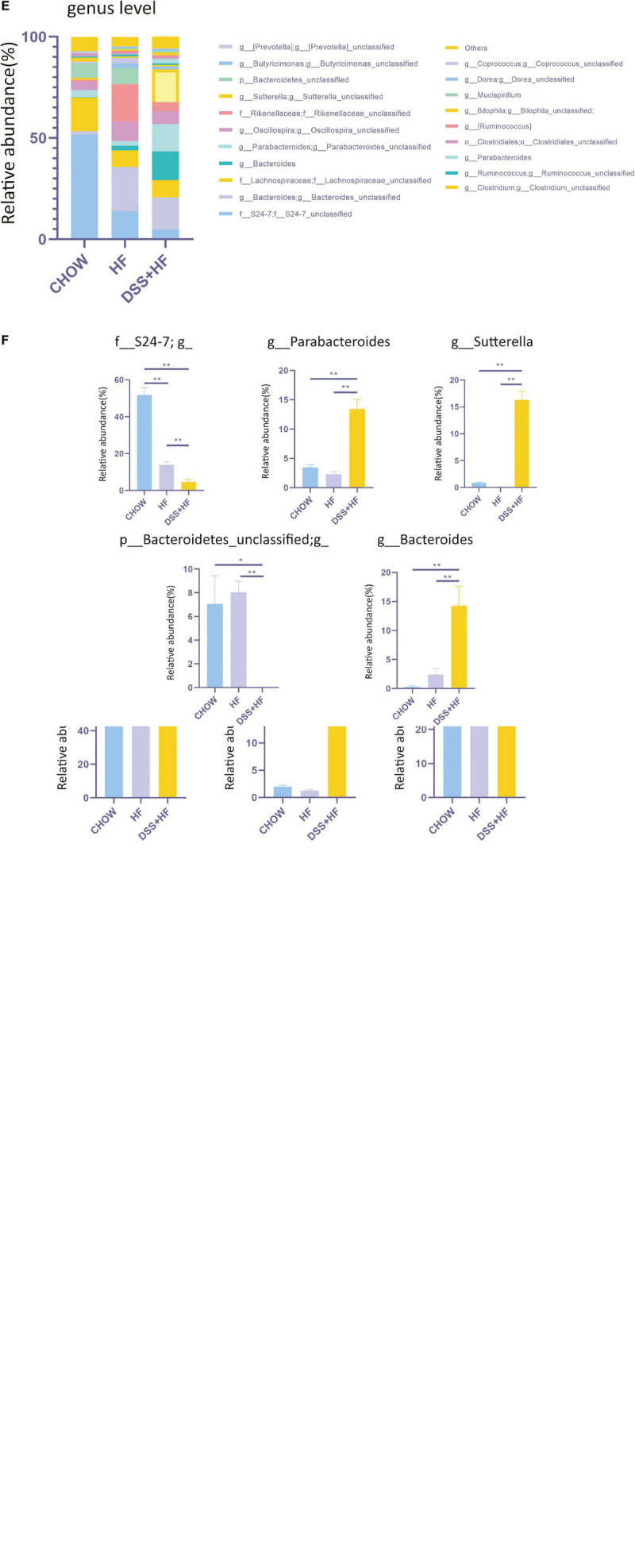
Community structure of gut microbiota shows distinct changes after DSS treatments. **(A)** The abundance of microbiota (Ace index of OTU) and the diversity of microbiota (Shannon index of OTU) in chow, HF, and DSS + HF group. The data are expressed as the mean ± SEM. * P < 0.05; ** P 0.01. **(B)** Bray–Curtis distance-based principal coordinate analysis (PCOA) for chow, HF, and DSS (+ HF group (9 per/group). **(C)** The phylum levels of microbiota in the above groups. **(D)** The representative phylum level distribution of microbiota in the above groups (9 per/group). The data are expressed as the mean ± SEM. The statistical analyses were done with Wilcoxon rank-sum test. NS, not statistically significant; *P < 0.05; **P < 0.01. **(E)** The genus levels of microbiota in the above groups. **(F)** The representative genus level distribution of microbiota in the above groups (9 per/group). The data are expressed as the mean ± SEM. The statistical analyses were done with Wilcoxon rank-sum test. *P < 0.05; **P < 0.01.

We investigated the diversity of the intestinal microbiota using 16S rRNA sequencing. No significant differences were observed between the HF and DSS + HF groups with respect to the abundance of microbiota, as indicated by the Ace index. However, the microbiota was more abundant in the HF and DSS + HF groups than in the chow group (Figure 3A). In addition, the α diversity of microbiota (Shannon index) was comparable between the HF and DSS + HF groups. However, the microbiota in the HF and DSS + HF groups was less diverse than that in the chow group (Figure 3A). These results showed that an HF diet may increase the abundance but decrease the diversity of the microbiota, which was not affected by DSS treatment.

However, Bray–Curtis distance-based PCOA of the gut microbiota composition showed a distinct deviation along PCOA1 for the HF and DSS + HF groups (explaining 19.5% of the variation) (Figure 3B), suggesting significant changes in the core microbiota after DSS treatment. Meanwhile, the gut microbiota composition in the chow and HF groups also showed a distinct deviation along PCOA2 (explaining 14.5% of the variation). Collectively, these results demonstrate that there were significant differences in the gut microbiota composition between the chow, HF, and DSS + HF groups.

Next, we explored the variations in the gut microbiota composition between chow, HF, and DSS + HF groups at both, the phylum and genus levels. At the phylum level, the microbiota was dominated by *p_Bacteroidetes*, *p_Firmicutes*, and *p_Proteobacteria* (Figure 3C). In the chow group, *p_Bacteroidetes*, *p_Firmicutes*, and *p_Proteobacteria* accounted for 65.4, 30.8, and 2.0% microbiota, respectively. Conversely, in the HF group, *p_Bacteroidetes*, *p_Firmicutes*, and *p_Proteobacteria* accounted for 71.3, 25.8, and 1.3% of microbiota, respectively. In the DSS + HF group, *p_Bacteroidetes*, *p_Firmicutes*, and *p_Proteobacteria* accounted for 56.8, 23.0, and 18.6% microbiota, respectively. In particular, *p_Proteobacteria* was significantly higher in the DSS + HF group than in the chow or HF groups (*p* < 0.01; Figure 3D). In addition, *p_Bacteroidetes* proportion was significantly higher in the HF group than in the DSS + HF group (*p* < 0.01; Figure 3D). However, there were no significant differences in the proportion of *p_Firmicutes* between the HF and DSS + HF groups (Figure 3D). In addition, *p_Actinobacteria* proportion was significantly higher in the DSS + HF group than in the HF (*p* < 0.05) or chow groups (*p* < 0.01). However, *p_Tenericutes* proportion was significantly lower in the DSS + HF group than in the HF (*p* < 0.05) or chow groups (*p* < 0.01; Supplementary Figure 2A).

The chow, HF, and DSS + HF groups also showed significant differences at the genus level (Figure 3E). The DSS + HF group had significantly higher proportions of *g_Parabacteroides* (*g_Parabacteroides*: DSS + HF vs. HF 13.4 vs. 2.3%; *p* < 0.01, DSS + HF vs. chow 13.4 vs. 3.5%, *p* < 0.01), *g_Sutterella* (*g_Sutterella*: DSS + HF vs. HF 16.3 vs. 0.007%; *p* < 0.01, DSS + HF vs. chow 16.3 vs. 0.92%, *p* < 0.01), and *g_Bacteroides* (*g_Bacteroides*: DSS + HF vs. HF 14.3 vs. 2.4%, *p* < 0.01; DSS + HF vs. chow 14.3 vs. 0.3%, *p* < 0.01) than the chow or HF groups. In contrast, the DSS + HF group had significantly lower proportions of *f_S24-7* (*f_S24-7* DSS + HF vs. HF 4.7 vs. 14.0%, *p* < 0.01; DSS + HF vs. chow 4.7 vs. 51.8%, *p* < 0.01) and *p_Bacteroidetes_unclassified* (*p_Bacteroidetes_unclassified* DSS + HF 0.005% vs. HF 8.0%, *p* < 0.01; DSS + HF 0.005% vs. chow 7.1%, *p* < 0.05) than the chow or HF groups (Figure 3F). In addition, the HF group had significantly higher proportion of *g_Rikenellaceae* (HF vs. chow 18.2 vs. 0.9%, *p* < 0.01; HF vs. DSS + HF 18.2 vs. 4.3%, *p* < 0.01) than the chow or DSS + HF groups (Supplementary Figure 2B).

### Dextran Sulfate Sodium Administration Alters Gene Expression Profiles of Non-parenchymal Cells

**FIGURE 4:**
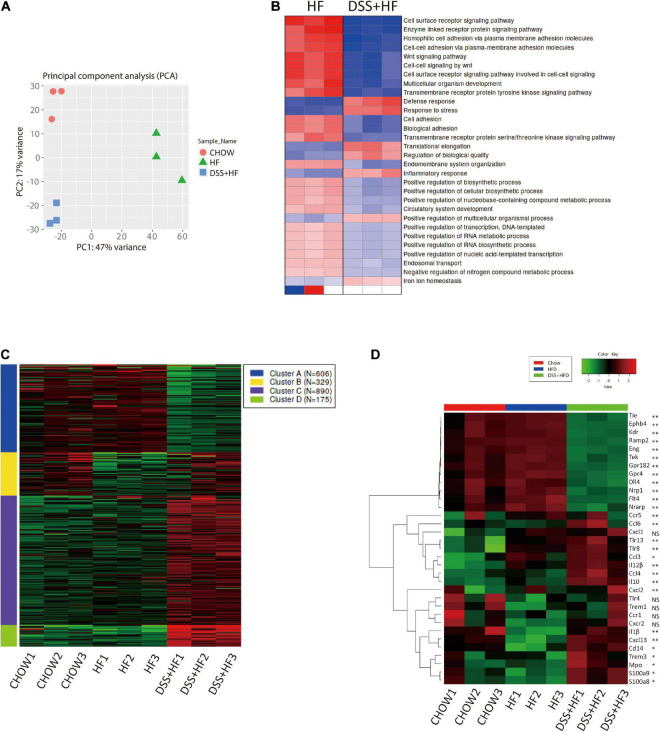
Gene expression profiles of liver non-parenchymal cells related to DSS treatment. **(A)** Principal component analysis (PCA) of chow, HF and DSS + HF group. **(B)** Heatmap of GO terms enriched for HF and DSS + HF groups. All *P* values in the GO enrichment analysis were adjusted for multiple testing using the BH method. Adjusted *P* < 0.05 was considered significant. **(C)** Genewise clustering heatmap of 2000 DEGs according to the gene expression patterns, showing segregation into four clusters. **(D)** Heatmap of the representative DEGs that are shared by Chow vs. HF vs. DSS + HF comparisons and enriched for “vascular and angiocrine signaling,” “Inflammatory response” and “Immune response.” DEG, differentially expressed gene. All *P* values were adjusted for multiple testing using the BH method. Adjusted *P* < 0.05 was considered significant. NS, not statistically significant; *P < 0.05; **P < 0.01.

Transcriptomic analysis of NPCs showed that the DSS + HF group had distinct gene expression signatures along PC1 (explaining 47% of variation) and PC2 (explaining 17% of variation) in comparison with the chow and HF groups (Figure 4A). In particular, the HF and DSS + HF groups displayed significantly different gene expression profiles. Compared with the control group, the number of differentially expressed genes (DEGs) in the HF and DSS + HF groups was 761 (403 upregulated and 358 downregulated) and 2,932 (1,666 upregulated and 1,266 downregulated), respectively. Notably, the HF and DSS + HF groups displayed significantly different trends, and 3,004 DEGs (1,607 upregulated and 1,397 downregulated) were identified between these two groups (Supplementary Figure 3A).

We also analyzed the sets of DEGs (compared with the chow group) using the Gene Ontology (GO) pathway enrichment analyses. We found that the DEGs between the HF and DSS + HF groups were mainly involved in the cell surface receptor signaling pathway, enzyme linked receptor protein signaling pathway, Wnt signaling pathway, cell–cell adhesion *via* plasma membrane adhesion molecules, multicellular organism development, defense response, inflammatory response, and response to bacteria. Interestingly, cell surface receptor signaling pathway, enzyme linked receptor protein signaling pathway, Wnt signaling pathway, cell-cell adhesion *via* plasma membrane adhesion molecules, and multicellular organism development were downregulated in the DSS + HF group, which was associated with the development of vasculature and angiocrine development. However, defense response, inflammatory response, and response to stress were upregulated in the DSS + HF group, reflecting the abnormal state of inflammatory response caused by DSS-induced colitis (Figure 4B).

**FIGURE 5:**
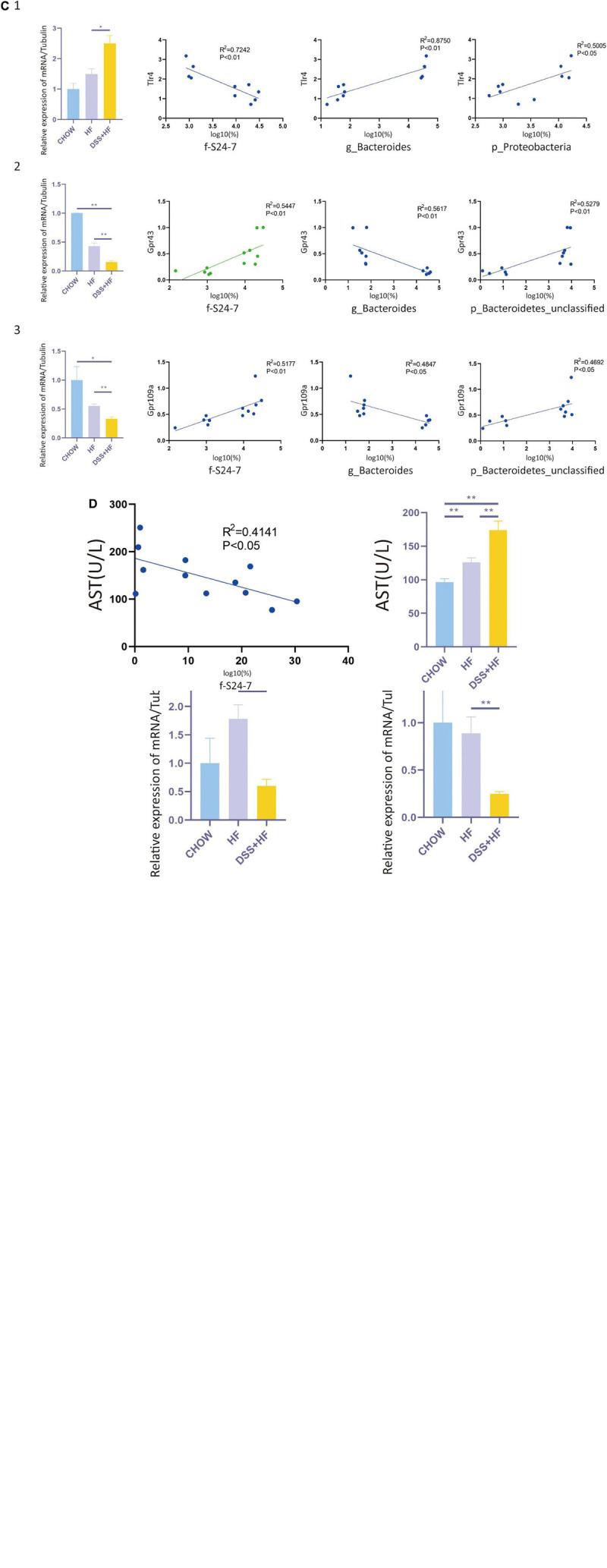
Correlation between gut microbiota and non-parenchymal cells (NPCs) gene expression. **(A-1)** Correlation between *p_Proteobacteria* and vascular development related genes (Dll4, Eng, and Gpr4). **(A-2)** Correla-tion between *p_Proteobacteria* and inflammation related genes (Ccl4, Cd14, S100a9, and Ccl6). **(A-3)** Correlation between *g_Bacteroides* and vascular development related genes (Dll4, Kdr, and Gpr4). **(A-4)** Correlation between *g_Bacteroides* and inflammation related genes (Ccl4, Ccl6, and Tlr13). **(A-5)** Correlation between *f_S24-7* and vascular development related genes (Dll4, Kdr and Gpr4). **(A-6)** Correlation between *f_S24-7* and inflammation related genes (Ccl4, Il12b, Tlr8, and Tlr13). X axis represents the percentage of gut bacterium in a logarithmic scale of base 10. Y axis represents the gene expression of NPCs in a logarithmic scale of base 10. **(B)** Cd31 and Dll4 expression at the RNA level in NPCs from the above groups. The statistical analyses were done with an unparied *t*-test. **P < 0.01. **(C-1)** Correlation between *f_S24-7*, *g_Bacteroides and p_Proteobacteria* and Tlr4. **(C-2)** Correlation between *f_S24-7, g_Bacteroides and p_Bacteroidetes* and Gpr43. **(C-3)** Correlation between *f_S24-7, g_Bacteroides and p_Bacteroidetes* and Gpr109a. X axis represents the percentage of gut bacterium in a logarithmic scale of base 10. **(D)** Correlation between *f_S24-7* and aspartate aminotransferase (AST). NS, not statistically significant; *P < 0.05; **P < 0.01.

Based on the gene expression profiles across all samples, we grouped the 2,000 identified genes among the three groups into four clusters (Figure 4C and Supplementary Figure 3B). These four clusters were functionally annotated using the GO enrichment analysis. We found that cluster A gene sets included genes such as, *Ramp2*, *Tek*, *Amotl1*, *Flt4*, *Sox17*, *Cdh13*, *Amotl2*, *Tie1*, *Ramp3*, *Mmrn2*, *Sox18*, *Nrarp*, *Nrp1*, *Epha2*, *Ccn2*, *Ptprb*, *Gata4*, *Abl1*, *Efna1*, and *Tgfa*, which were mainly associated with vasculature development, cell adhesion-related terms, and angiogenesis. Notably, compared with the chow and HF groups, NPCs derived from the DSS + HF group exhibited downregulated expression of the cluster A genes, such as those involved in vasculature development and angiogenesis. In particular, expression levels of genes coding for Notch1 and vascular endothelial growth factor (VEGF) receptor (*Dll4*, *Kdr*, *Flt4*, *Nrp1*, and *Nrarp*), TGF-β receptor (*Eng*), Ephrin B receptor (*Ephb4*), and receptor tyrosine kinase (*Tek* and *Tie1*), which are all membrane receptors for ligands important for vascular development and homeostasis, were significantly downregulated in the DSS + HF group (Figure 4D). The expression of *Dll4* as well as *Cd31* was further validated by quantitative real time polymerase chain reaction (qRT-PCR), which showed that the expression levels of both genes were significantly downregulated in the DSS + HF group (*Dll4*: DSS + HF 2.6 vs. chow 1.3, DSS + HF 0.3 vs. HF 0.9; *p* < 0.05) (*Cd31*, DSS + HF 2.6 vs. chow 1.3; DSS + HF 0.6 vs. HF 1.8, *p* < 0.01; Figure 5B).

Cluster B was enriched in terms associated with the immune system and inflammatory response (*Ifitm1*, *Ccr1*, *Cxcr2*, *Xcl1*, *Lat*, *Ccl17*, *Thy1*, *Ccl5*, and *e*, among others). Enriched terms for cluster C genes were also related to immune system and inflammatory responses, such as fatty acid metabolic processes (*Ccl3*, *Prdx2*, *Hmox1*, *Cybb*, *Ccl6*, *Ccl4*, *Ccl9*, *Cpb2*, *Kng1*, and *Vegfa*, among others) and carbohydrate transport (*Slc2a2*, *Slc2a5*, *Slc2a7*, *Slc5a1*, *Slc5a4*, *Slc5a9*, *Aqp1*, *Aqp7*, and *e*, among others). Cluster D gene sets were mainly enriched in immune response (*Elane*, *Btnl10*, *Cxcl13*, *Apcs*, *Ppbp*, *Pf4*, *Camp*, *Fpr2*, *Ifitm6*, *Fgr*, *Fcrl5*, *Trim10*, *Klrb1b*, *Bcl2a1d*, *Pparg*, *Cd244a*, *Il10*, and *Cxcl14*, among others) and responses to bacteria (*Nr1h3*, *Mpo*, *Cxcl13*, *Ppbp*, *Pf4*, *Camp*, *Epx*, *Il10*, *Wfdc21*, *Elane*, *Fos*, *Snca*, *Lcn2*, *Prg2*, *Fga*, *Hp*, *Ltf*, *Fgb*, *Isg15*, and *Ctsg*, among others). Compared with the HF group, expression levels of cluster B, C, and D genes in the DSS + HF group were significantly increased. Expression of chemokines (*Ccl3, Ccl4*, and *Ccl6*), chemokine receptors (*Ccr1, Ccr5, Cxcr2, Cxcl1, Cxcl2*, and *Cxcl13*), and inflammatory factors (*Mpo*, Il-1β, and IL12) increased significantly in the DSS + HF group (Figure 4D). In addition, expression levels of toll-like receptors (*Tlr8* and *Tlr13*) and CD14 were upregulated in the DSS + HF group (Figure 4D). Furthermore, expression levels of S100A8 and S100A9 were upregulated in the DSS + HF group (Figure 4D). These genes are abundantly expressed by immune cells, such as monocytes and macrophages (Gebhardt et al., 2006). Additionally, expression of *Trem1*, which is an amplifier of the inflammatory responses triggered by bacterial and fungal infections, was also increased in the DSS + HF group. *Trem1* expression can stimulate neutrophil and monocyte-mediated inflammatory responses and trigger the release of pro-inflammatory chemokines and cytokines. *Ccl3* (macrophage inflammatory protein-1 α, MIP-1α) expression was also significantly increased in the DSS + HF group.

### Correlation Between Gut Microbiota Abundance and Non-parenchymal Cell Gene Expression

Next, we investigated whether there was a correlation between NPC gene expression and gut microbiota abundance. We found that the abundance of Proteobacteria correlated negatively with vascular development and expression of homeostasis-related genes, such as *Dll4*, *Kdr*, *Nrp1*, *Eng*, *Ephb4*, *Tek*, *Tie1*, and *Gpr182*. In contrast, *p_Proteobacteria* correlated positively with the expression levels of *e* and *Ccl6*, which are inflammation-related genes (Figure 5A). Abundance of the *g_Bacteroides* correlated negatively with vascular development and expression of homeostasis-related genes, such as *e* and *Gpr4*. The abundance of *g_Bacteroides* correlated positively with the expression of inflammation-related genes, such as *Ccl6*, *Ccl4*, and *Tlr13* (Figure 5A). The abundance of *f_S24-7* correlated positively with vascular development and expression of homeostasis-related genes, such as *Dll4*, *Kdr*, and *e*. The abundance of *f_S24-7* correlated negatively with the expression of inflammation-related genes, such as *Ccl4, Il12b, Tlr*8, and *Tlr13* (Figure 5A).

As *g_Proteobacteria* was related to the production of LPS, whereas *f_S24-7*, *g_Bacteroides*, and *p_Bacteroidetes* were related to the production of short chain fatty acids (SCFAs), we further examined the expression of SCFAs receptors, such as GPR41, GPR43, and GPR109A, and that of toll like receptor 4 (TLR4), which is the receptor of LPS. The expression level of *Tlr4*, as detected by qPCR analysis, significantly increased in the DSS + HF group (Figure 5C) (DSS + HF vs. chow; *p* < 0.01; DSS + HF vs. HF; *p* < 0.01). The correlation between *Tlr4* expression and the abundance of *p_Proteobacteria*, *g_Bacteroides*, and *f_S24-7* was analyzed (Figure 5C). The abundances of *p_Proteobacteria* and *g_Bacteroides* correlated positively with the expression of *Tlr4*. In contrast, the abundance of *f_S24-7* correlated negatively with the expression of *Tlr4*. However, the expression of *Gpr43* and *Gpr109a*, as detected by qPCR, decreased significantly in the DSS + HF group (Figure 5C) (DSS + HF vs. HF; *p* < 0.05). Meanwhile, the abundance of *g_Bacteroides* correlated negatively with the expression of *Gpr43* and *Gpr109a*. In contrast, the abundance of *f_S24-7* and *p_Bacteroidetes* showed a positive correlation with the expression of *Gpr43* and *Gpr109a* (Figure 5C). Furthermore, the abundance of *f_S24-7* correlated negatively with the level of aspartate aminotransferase (AST), which is an important clinical indicator of liver injury (*R*^2^ = 0.4141, *p* < 0.05; Figure 5D).

## Discussion

Although the incidence of NAFLD is high in patients with IBD, and the gut microbiota plays an important role in both diseases, the relationship between IBD and NAFLD and the specific role of microbiota dysbiosis remain unclear. Studies have shown that an HF diet promotes the development of NASH and gut microbiota dysbiosis, which can be enhanced by DSS-induced colitis. However, previous studies have not investigated the role of DSS-induced colitis mediated gut microbiota dysbiosis in aggravating NASH. Recent studies have shown that in addition to genetic susceptibility and diet, the gut microbiota affects hepatic glycolipid metabolism and the balance between proinflammatory and anti-inflammatory effectors in the liver (Kolodziejczyk et al., 2019). It has been suggested that dysbiosis of the gut microbiota can be an important factor in the development of NASH (Buzzetti et al., 2016). A correlation between NAFLD-associated inflammation and the abundance of bacteria has been observed in both, patients with NASH and animal models (Kolodziejczyk et al., 2019). The abundance of *p_Proteobacteria*, *g_Bacteroides*, *g_Enterobacteria*, and *g_Escherichia* is associated with the NAFLD progression (Le Roy et al., 2013; Zhu et al., 2013; Boursier et al., 2016). However, the mechanisms by which the gut microbiome modulates NASH development remain unknown. It has been suggested that the gut microbiota and their products, such as LPS and SCFAs, translocate to the liver upon the gut barrier disruption, and participate in the modulation of NASH (Kolodziejczyk et al., 2019). In this study, we focused on the role of DSS-induced colitis dependent gut microbiota dysbiosis in NASH development, and assessed the correlation between the expression of liver inflammation-related genes and the abundance of gut bacteria. A 60% HF diet was used to establish a mouse model of NASH. DSS treatment (1%) was used to induce colitis in cycles, and each cycle consisted of 7 days of DSS administration followed by a 10-day interval with normal drinking water, which was consistent with previous studies (Gäbele et al., 2011; Cheng et al., 2018). Jelena et al. have shown that fructose can disrupt the intestinal mucosal barrier leading to the leak of endotoxins into the liver, thereby promoting NASH development (Todoric et al., 2020). Therefore, we combined a high-fructose diet with an HF diet to establish a NASH model. The cycles were repeated for 19 weeks, to achieve a severe NASH phenotype. We found that compared with the HF group, DSS-induced colitis enhanced the disruption of the intestinal mucosal barrier and increased the permeability of the vascular barrier, further aggravating liver inflammation and fibrosis in the NASH model. Moreover, this effect was accompanied with dysbiosis of the gut microbiota.

The abundance of *p*_*Proteobacteria* increased significantly in the NASH model with DSS-induced colitis. Studies have shown that the gram-negative *p*_*Proteobacteria* contain large amounts of LPS in their outer membrane, which can play an important role in the development of liver inflammation and fibrosis (Rizzatti et al., 2017; Carpino et al., 2020). The increased abundance of *p*_*Proteobacteria* observed in our study may increase the levels of LPS, which may in turn aggravate liver inflammation and fibrosis. SCFAs are important metabolic products of several gut bacteria. Although most SCFAs are utilized in the gut, some may be transported into the liver *via* the portal vein and play an important role in the development of NASH. SCFAs limit the development of NASH *via* downregulating inflammatory signals and enhancing insulin sensitivity (Kolodziejczyk et al., 2019). We found that DSS-induced colitis significantly changed the abundance of *p*_*Bacteroidetes*, *f_S24-7*, and *g_Bacteroides.* The *p*_*Bacteroidetes* is a major source of propionate and can suppress inflammation (Parada Venegas et al., 2019). The *f_S24-7*, which belongs to the *p*_*Bacteroidetes*, can also deconstruct complex carbohydrates and promote SCFA production (Mefferd et al., 2020). However, *g_Bacteroides* correlated negatively with the amount of SCFAs. Boursier et al. (2016) showed that the abundance of *g_Bacteroides* is increased significantly in patients with NASH. In our study, the decreased abundance of *p_Bacteroidetes* and *f_S24-7* and the increased abundance of *g_Bacteroides* implied that DSS-induced colitis may decrease the amounts of SCFAs in gut. This decrease in the SCFAs may restrict their ability to limit the progression of liver inflammation and fibrosis. In addition, there was an increase in the abundance of *g_Parabacteroides* and *g*_*Sutterella* in the DSS + HF group. A previous study revealed that patients with NASH have a higher abundance of *g_Parabacteroides*, suggesting that *g_Parabacteroides* is associated with the development of NASH (Wong et al., 2013). Moreover, *g*_*Sutterella* has been reported to impair the functionality of the intestinal antibacterial immune response, and importantly, interfere with its capacity to limit intracellular bacterial species (Kaakoush, 2020). Thus, we propose that the DSS dependent disruption of the intestinal barrier may have been a consequence of the increased abundance of *g_Sutterella.*

Our analysis of gene expression patterns in NPCs showed that genes associated with the immune system, inflammatory response, and vasculature development were differentially expressed between the DSS + HF and HF groups. Previous studies have reported on the disruption of vascular and angiocrine signaling in mice and humans during NASH pathogenesis (Xiong et al., 2019). Our results showed that the expression of angiocrine signaling-related genes, such as *Cd31* and *Dll4*, was significantly decreased in the DSS + HF group, further implying that DSS-induced colitis enhanced the disruption of angiocrine signaling in the liver. Furthermore, inflammatory pathways were activated in liver NPCs of the DSS + HF group. *e* is an amplifier of inflammatory responses triggered by bacterial and fungal infections and can stimulate neutrophil and monocyte-mediated inflammatory responses (Jérémie et al., 2015). *Trem1* expression is upregulated in mice with DSS-induced colitis. In addition, DSS treatment increased the expression of S100A8 and S100A9, which was expressed abundantly in immune cells, such as monocytes and macrophages (Gebhardt et al., 2006). Accordingly, we observed that the expression levels of genes encoding chemokines (*Ccl3*, *Ccl4*, and *Ccl6*), chemokine receptors (*Ccr1*, *Ccr5*, *Cxcr2*, *Cxcl1*, *Cxcl2*, and *Cxcl13*), and inflammatory factors (*Mpo*, Il-1β, and Il12) were increased in mice with DSS-induced colitis. In addition, toll-like receptors (*Tlr4*, *Tlr8*, and *Tlr13*) and CD14 expressions were upregulated in the DSS + HF group. These results suggest the activation of macrophage-mediated inflammatory responses. Furthermore, we also found that the proportion of macrophages was increased in mice with DSS-induced colitis, which may further enhance the inflammatory responses mediated by macrophages.

Although the abundance of *p_Proteobacteria* and *g_Bacteroides* is higher in patients with NASH (Zhu et al., 2013; Boursier et al., 2016), the correlation between the abundance of microbiota and expression of inflammation-related genes remains unclear. Our results indicated that the abundance of *p_Proteobacteria* and *g_Bacteroides* correlated positively in NPCs with genes related to inflammation. In contrast, the abundance of *f_S24-7* correlated negatively with the expression of inflammation-related genes in NPCs. The disruption of the intestinal barrier and increased gut-vascular permeability can lead to the translocation of the gut microbiome and its products, such as LPS into the liver. TLR4 and its co-receptor CD14 can be activated by LPS, and thereafter trigger a downstream inflammatory cascade in the liver (Kolodziejczyk et al., 2019). Thus, the increased abundance of *p_Proteobacteria* and *g_Bacteroides* may lead to increased levels of LPS and cause an upregulation in the expression of *Tlr4*, *Cd14*, and the downstream inflammatory genes (*Mpo* and Il-1β, among others). Additionally, we found that the abundance of SCFA-producing bacteria, such as *p*_*Bacteroidetes* and *f*_*S24-7*, correlated positively with the expression of SCFAs receptors, such as GPR43 and GPR109A, in NPCs. The decreased expression of GPR43 and GPR109A may result in defective SCFA-mediated anti-inflammatory responses. Our results indicated that upregulated expression of genes related to inflammation may be the consequence of changes in bacterial composition, especially in LPS- and SCFA-producing bacteria. Given the important role of SCFA-producing bacteria in the abundance of *f_S24-7* correlated negatively with the levels of AST, implying that *S24-7* could be a marker for liver inflammation.

**FIGURE 6:**
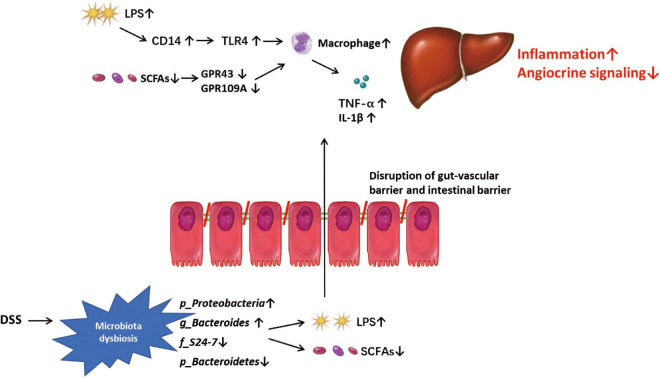
Mode pattern. DSS-induced microbiota dysbiosis, especially the increased lipopolysaccharide (LPS)-pro-ducing microbiota and the decreased SCFAs-producing microbiota, might play a role in disrupting the gut-vascular barrier and inducing more LPS into liver, and promote the disease progression in liver through triggering macrophage-mediated inflammatory response and disrupting angiocrine signaling.

In summary, our study shows that DSS-induced colitis can disrupt the gut-vascular barrier and aggravate hepatic inflammation and fibrosis in mice with NASH. DSS-induced microbiota dysbiosis, especially an increase in LPS-producing (*g*_*Proteobacteria*) bacteria and decrease in SCFA-producing bacteria (*p*_*Bacteroidetes*, *f_S24-7* and *g_Bacteroides*) may disrupt vascular and angiocrine signaling and promote the expression of genes related to inflammation in NPCs, thereby promoting liver inflammation and fibrosis (Figure 6). In addition, the abundance of *f_S24-7* in the gut microbiota may be a potential marker for the progression of NASH, which must be investigated in future studies.

## Data Availability Statement

The datasets presented in this study can be found in online repositories. The names of the repository/repositories and accession number(s) can be found below: https://www.ncbi.nlm.nih.gov/bioproject; PRJNA756374.

## Ethics Statement

The animal study was reviewed and approved by Animal Ethics Committee of Shanghai First People’s Hospital.

## Author Contributions

BS, JW, YG, TG, ZS, CZ, BL, XX, FL, and QZ performed the experiments and analyzed the data. LL, XC, and HD designed the study. BS, HD, and LL wrote the manuscript. XC revised the manuscript. All authors read and approved the final manuscript.

## Conflict of Interest

The authors declare that the research was conducted in the absence of any commercial or financial relationships that could be construed as a potential conflict of interest.

## Publisher’s Note

All claims expressed in this article are solely those of the authors and do not necessarily represent those of their affiliated organizations, or those of the publisher, the editors and the reviewers. Any product that may be evaluated in this article, or claim that may be made by its manufacturer, is not guaranteed or endorsed by the publisher.

## Footnotes

1
http://bioinformatics.sdstate.edu/idep/
