# Supplementary figures and images for: Dextran Sulfate Sodium Salt-Induced Colitis Aggravates Gut Microbiota Dysbiosis and Liver Injury in Mice With Non-alcoholic Steatohepatitis
Source: Front Microbiol. 2021 Nov 2;12:756299. doi: 10.3389/fmicb.2021.756299 (PMC8593467; doi:10.3389/fmicb.2021.756299)

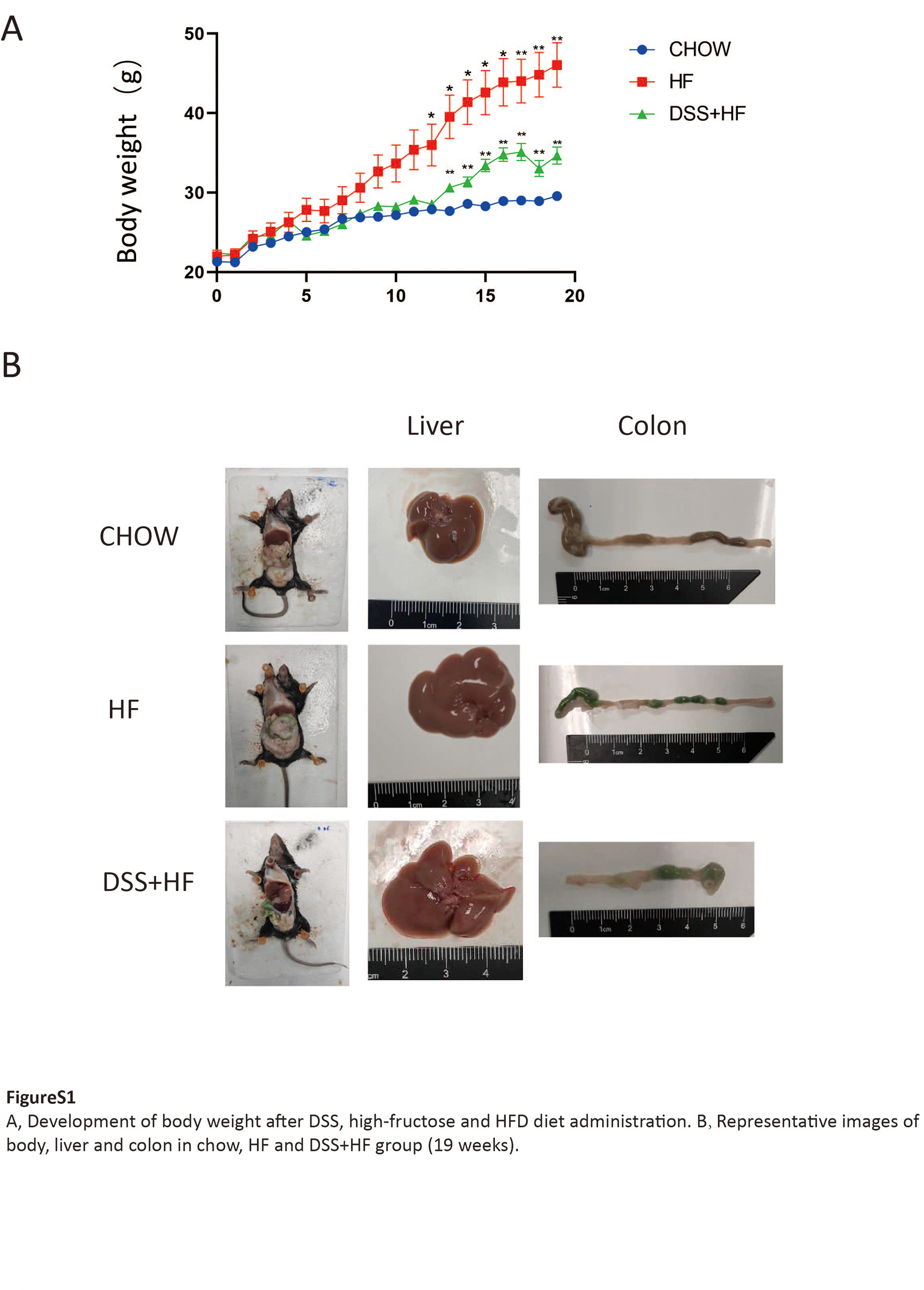

Supplement: Supplementary file 1 [file Image_1.JPEG]

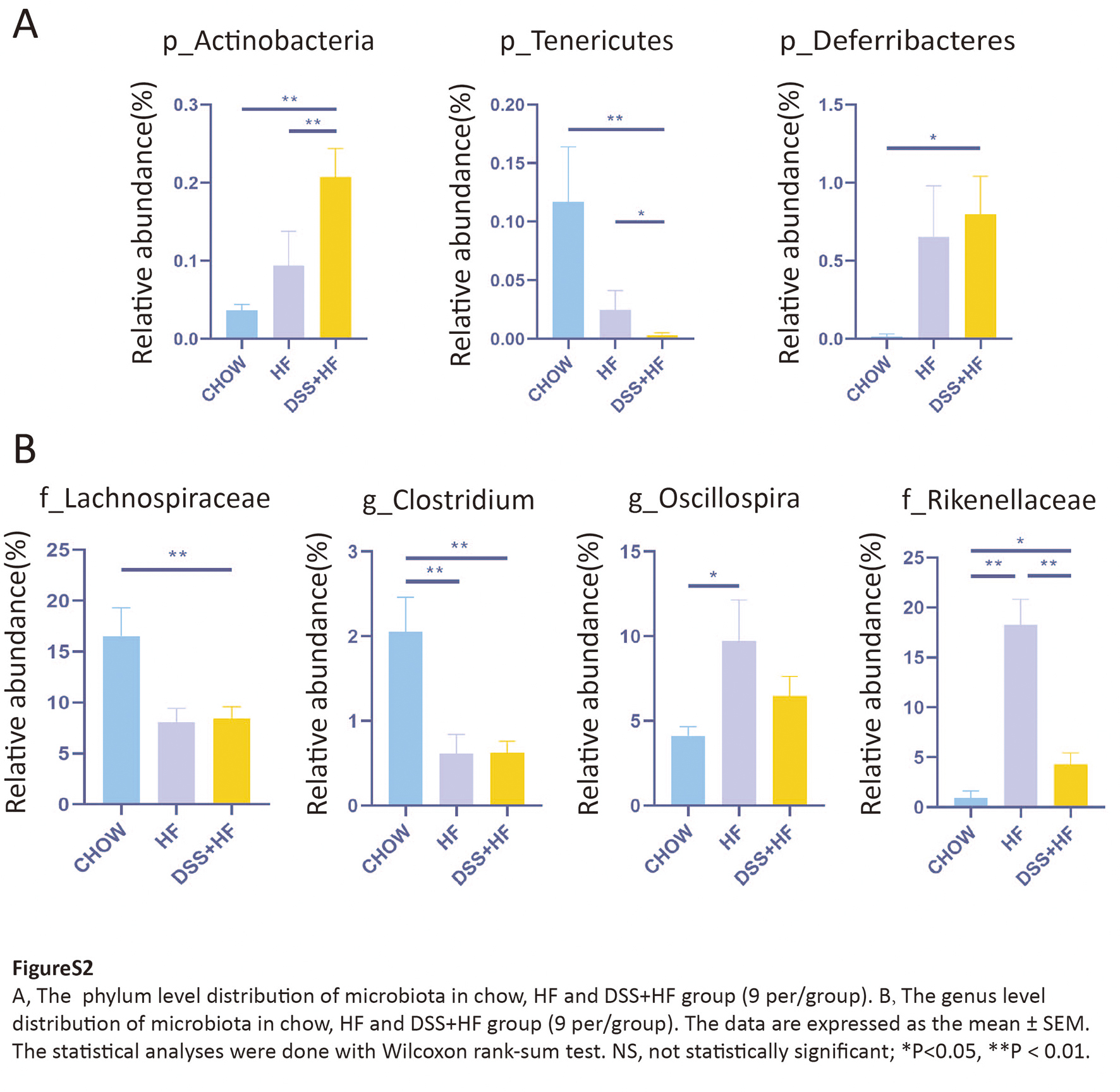

Supplement: Supplementary file 2 [file Image_2.JPEG]

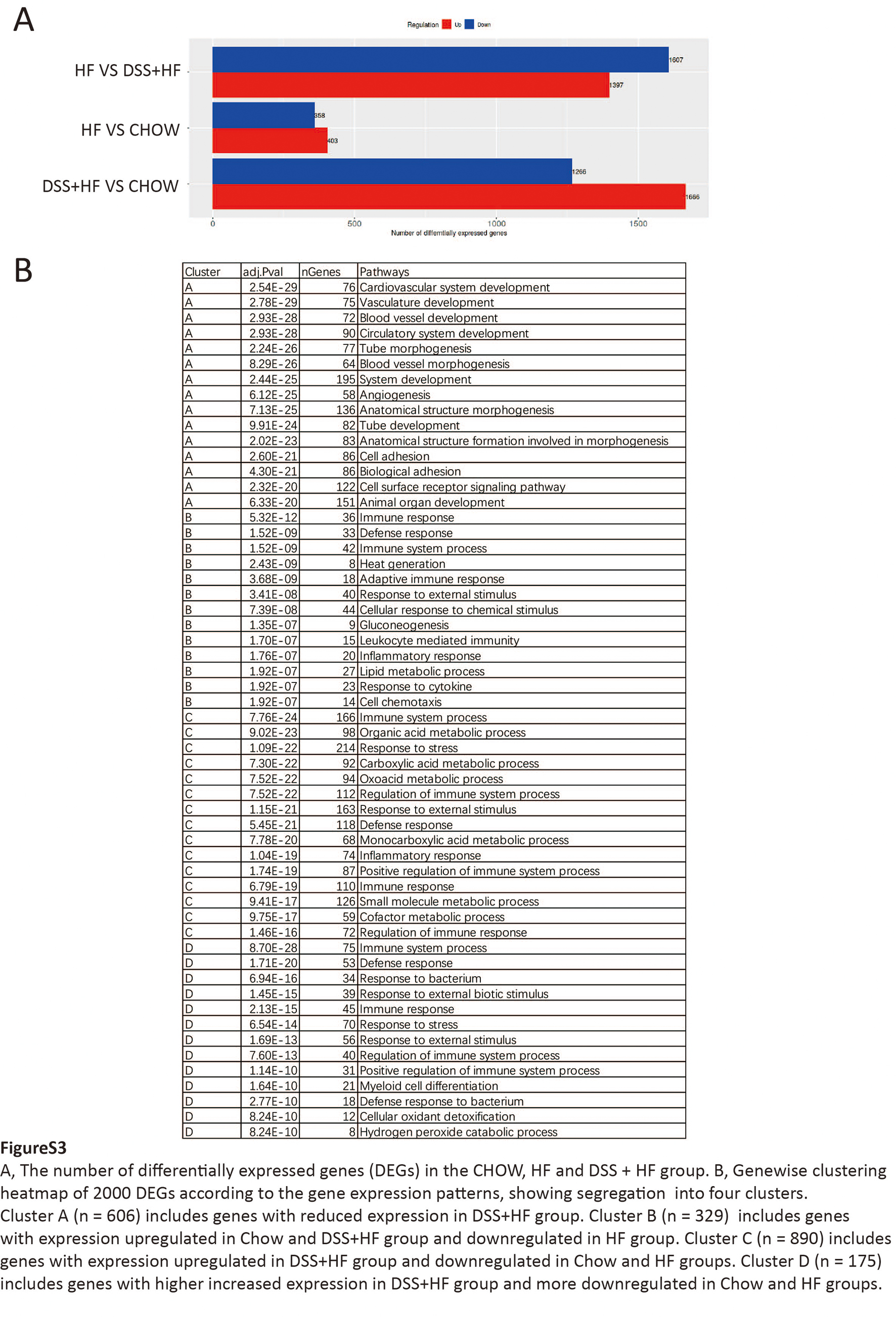

Supplement: Supplementary file 3 [file Image_3.JPEG]

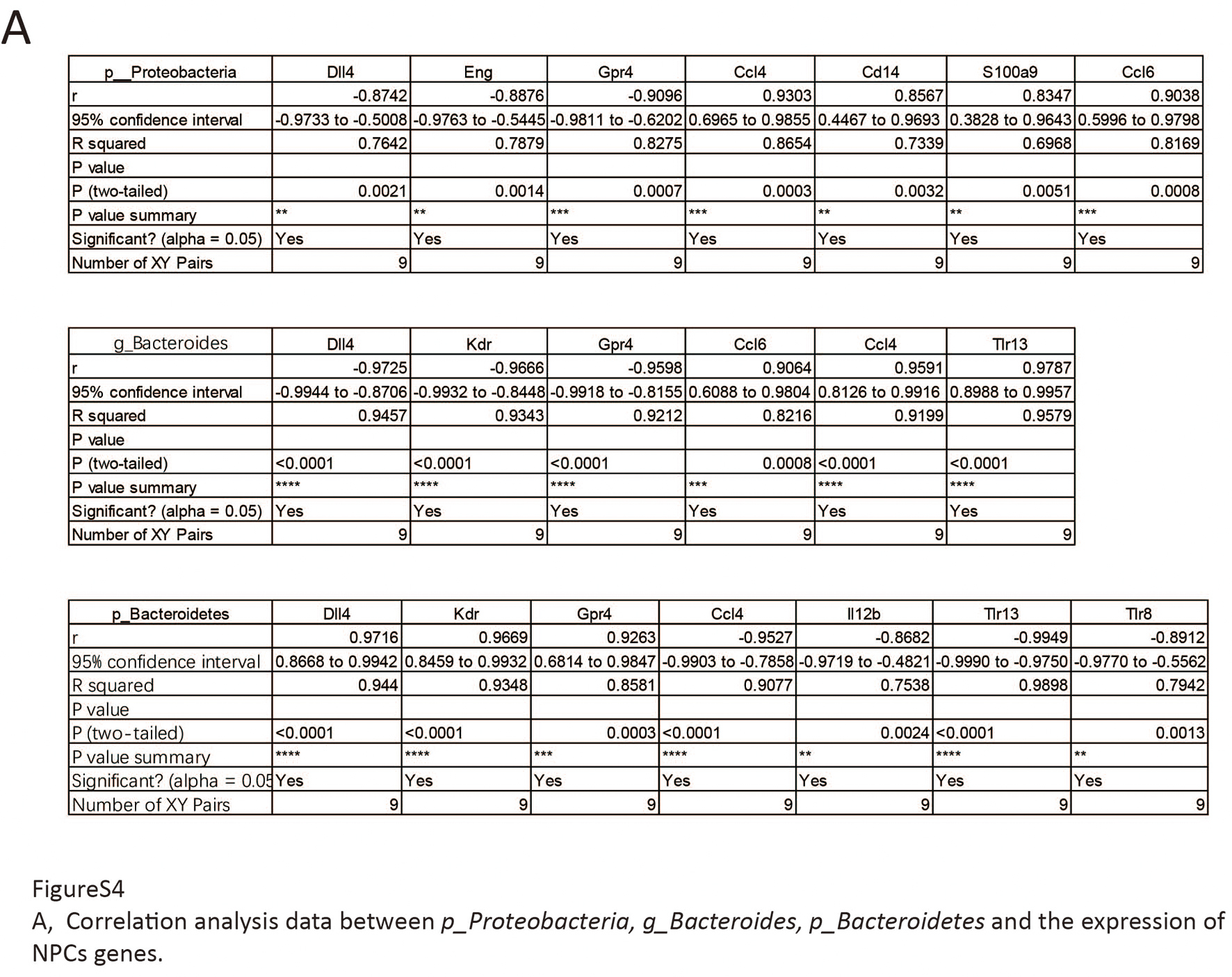

Supplement: Supplementary file 4 [file Image_4.JPEG]
